# Hypertriglyceridemia in New-Onset Type 1 Pediatric Diabetes

**DOI:** 10.1155/pedi/8425032

**Published:** 2025-06-26

**Authors:** Colleen A. Macke, Iman Al-Gadi, Nidhi Bansal, Sarah K. Lyons, Aikaterini A. Nella

**Affiliations:** Division of Diabetes and Endocrinology, Texas Children's Hospital and Baylor College of Medicine, Houston, Texas, USA

**Keywords:** hypertriglyceridemia, lipid, new-onset diabetes, pediatric, type 1 diabetes

## Abstract

Hypertriglyceridemia (HTG) in the setting of newly diagnosed diabetes is common in both adult and pediatric populations, as insulin deficiency promotes lipolysis and impairs triglyceride (TG) clearance. Severe HTG (defined as TG levels above 1000 mg/dL) in pediatric patients with new-onset type 1 diabetes mellitus (T1D) is rare; the true incidence and sequela of this phenomenon have not been well characterized. We present a single-center experience on severe HTG in pediatric patients with new-onset T1D between 2013 and 2022 and summarize the cases previously reported in the literature. Our cases display variability in their presentation and in their association with high-risk complications, such as acute pancreatitis. We discuss suggestions of early screening for HTG and pancreatitis in patients with protracted abdominal pain, and close monitoring of those identified to have significant HTG.

## 1. Introduction

Insulin deficiency in diabetes can result in hypertriglyceridemia (HTG), as insulin is an anti-lipolytic agent and regulator of lipoprotein lipase (LPL) enzyme actions [1]. HTG is common in new-onset diabetes in both adult and pediatric populations [2]. Severe and very severe HTG, defined as triglyceride (TG) levels above 1000 and 2000 mg/dL, respectively, are considered rare in the setting of new-onset diabetes with or without diabetic ketoacidosis (DKA) and are associated with absolute insulin deficiency [3, 4]. The incidence of severe HTG in new-onset type 1 diabetes (T1D) is unknown, with limited reported cases in the pediatric literature [5–21]. It is recognized that proper management of HTG in diabetes is critical to mitigate the risk of acute pancreatitis and its associated complications. We report a single-center experience of five cases of new-onset T1D in pediatric patients with severe HTG seen between 2013 and 2022. We discuss the patients' clinical presentation and management challenges. We also provide a succinct review of similar cases previously reported in the literature and recommendations for screening.

## 2. Case A

A 16-year-old previously healthy male presented to the emergency department (ED) with severe HTG, identified on the pediatrician's screening lipid profile (TG of 8486 mg/dL and > 10,000 mg/dL in two separate samples). Preceding symptoms included fatigue, polyuria, polydipsia, and a 10.5 kg weight loss, the latter previously attributed to initiation of methylphenidate therapy for attention deficit hyperactivity disorder. His BMI at time of presentation was at the 22^nd^ percentile. Family history was notable for dyslipidemia in first- and second-degree relatives and premature coronary artery disease (maternal grandmother with myocardial infarction at age 40). ED labs revealed hyperglycemia (blood glucose 354 mg/dL) and ketosis (beta-hydroxybutyrate 7 mmol/L) without severe acidosis (pH: 7.34, bicarbonate: 18 mmol/L). Islet cell autoantibodies, glutamate-decarboxylase (GAD) autoantibodies, and thyroid peroxidase antibodies were positive; stimulated c-peptide and insulin levels were low, indicating type 1 diabetes mellitus (T1D). Hemoglobin A1c (A1c) was 10.5%, thyroid stimulating hormone was 3.11 mIU/L and lipase was 172 U/L. Physical exam was remarkable for dry mucous membranes. He was fluid resuscitated and placed on a low-fat diet and subcutaneous insulin injections every 3 h.

**Figure 1:**
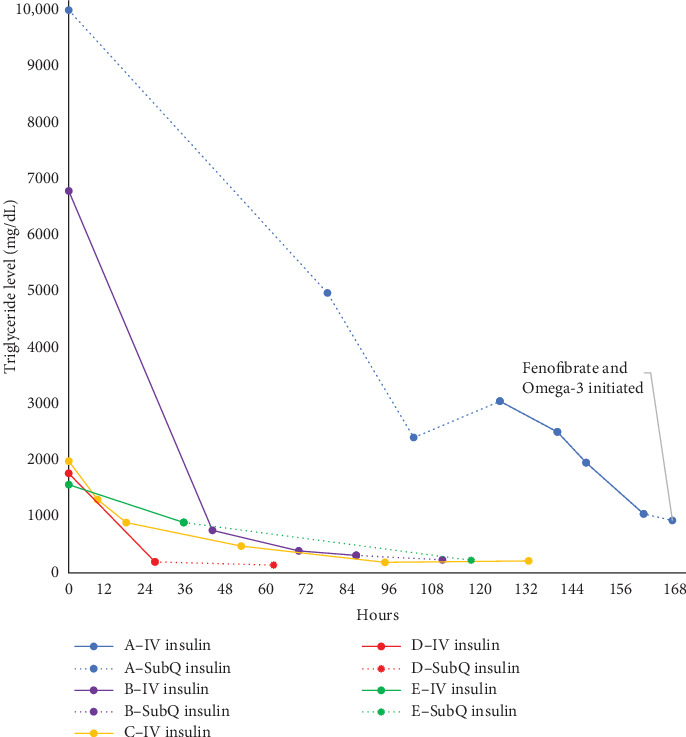
Serum triglyceride trends in relation to therapy during hospitalization.

Serial TG levels obtained every 12 h showed a progressive TG decline over the first 36 h of hospitalization on an average subcutaneous insulin dose of 1.3 units/kg/day (Figure 1). He had rebound HTG to 3046 mg/dL on day 3 of admission, with no significant hyperglycemia or ketosis. IV insulin therapy (0.1 units/kg/h) combined with fasting was initiated at that time and continued for 30 h until TG declined to 1042 mg/dL.

Two days after discharge, the TG level remained stable at 925 mg/dL. Following consultation with a lipidologist and exclusion of secondary causes of HTG, fenofibrate (120 mg daily), and omega-3 acid ethyl esters (1 g twice daily) were initiated due to concern for familial HTG. The fenofibrate dose was decreased 1 month later to 45 mg daily based on a TG level of 81 mg/dL, but the omega-3 acid dose (2 g daily) remained unchanged. Sixteen months later, his TG level was 120 mg/dL on fenofibrate and omega-3 acid. Diabetes was well controlled at that time with an A1c of 6.4%. Genetic testing for familial HTG has not yet been performed.

## 3. Case B

A 7-year-old male with autism spectrum disorder presented to the ED in DKA due to new-onset diabetes. Preceding symptoms included a 6-month history of polyuria, polydipsia, nocturia, and weight loss. Initial exam was notable for Kussmaul respirations, tachycardia, and dry mucous membranes. Abdominal exam was unremarkable. BMI and weight were below the 1st and 2nd percentiles for age and sex, respectively. Lab results showed severe DKA (Glucose: 412 mg/dL, bicarbonate: < 5 mmol/L, pH: 6.94, and serum beta-hydroxybutyrate: 11 mmol/l), with acute kidney injury (creatinine: 1.37 mg/dL). A1c was 13.5%. Lipase level was 354 U/L. He received fluid resuscitation and IV insulin therapy (0.1 units/kg/h). In the pediatric intensive care unit (PICU), he developed altered mental status and was treated with hypertonic saline for suspected cerebral edema. His neurological status improved and he returned to baseline within 12 h of hospitalization. Lipid labs obtained by the pediatrician 1 day prior to ED presentation resulted with severe HTG (6784 mg/dL). Family history was notable for hyperlipidemia in 1 second-degree relative and myocardial infarction before age 30 in a great-grandparent.

The patient remained fasting and on IV insulin infusion for a total of 43 h; was kept on IV insulin past DKA resolution (at 28 h—DKA resolution defined as bicarbonate >15 mmol/L at our institution) to facilitate TG level reduction to < 500 mg/dL (Figure 1). TG levels continued to decline after transition to subcutaneous insulin, reaching 225 mg/dL on the day of discharge (64 h after presentation). He tested positive for GAD autoantibodies, his stimulated c-peptide and insulin were low, consistent with T1D. Thyroid autoantibodies were negative and TSH was within normal limits (1.28 uIU/mL). At the 3-month follow-up with a lipidologist, TG were normal and without additional therapy. Repeat TG levels at 7 months and 32 months after initial presentation remained normal.

## 4. Case C

A 15-year-old male with history of prematurity, spastic quadriplegia, autism, developmental delay (nonverbal) and Graves' disease refractory to radioactive iodine ablation presented to the ED in mixed DKA and hyperosmolar hyperglycemic state (HHS) due to new-onset diabetes. Outpatient labs 2 weeks prior to presentation showed hyperglycemia (glucose: 319 mg/dL). He arrived to the ED lethargic and noncommunicative (Glasgow Coma Scale of 10) with Kussmaul respirations, fever, and emesis. BMI was at the 35^th^ percentile. Initial lab results revealed hyperosmolar DKA with glucose 1813 mg/dL, pH 7.12, lactic acid 6.3, beta-hydroxybutyrate 6.69 mmol/L, elevated creatinine (1.75 mg/dL), HTG (TG 1981 mg/dL), and lipase elevation (806 U/L). Insulin level was 3.5 mU/L, and c-peptide level 1.05 ng/mL. He developed hypotension and respiratory failure in the PICU, requiring mechanical ventilation and vasopressors, presumed due to DKA/HHS. He had rapid hyperglycemia correction with IV fluids alone; IV insulin (starting rate 0.05 units/kg/h) was added ~24 h after arrival to the hospital and titrated up to a maximal rate of 0.23 units/kg/h. He was managed for pancreatitis based on his degree of lipase elevation. Abdominal exam was limited due to degree of sedation needed for line protection and abdominal imaging was not felt to be necessary as treatment for presumed pancreatitis was already underway [22]. TG levels normalized by hospital day 4. He remained fasting on bowel rest to manage pancreatitis, so IV insulin infusion was continued to maintain tight glycemic control until hospital day 9 when feeds resumed. No repeat TG level was obtained after transition to subcutaneous insulin. There was a family history of autoimmune disease with lupus in the patient's mother, but no family history of hyperlipidemia.

Although his diabetes workup showed negative diabetes autoantibodies (GAD, insulin, CA 152, and zinc transporter autoantibodies), he was classified as antibody-negative type 1b based on his severe presentation, personal history of autoimmune thyroid disease, and thin body habitus. Patient had transient recovery of pancreatic function and was able to wean off insulin temporarily; 18 months later, he resumed multiple daily injections of insulin. TG level has not yet been rechecked.

## 5. Case D

A previously healthy 6-year-old female presented to the ED in DKA due to new-onset T1D. Preceding symptoms included 1 month of progressive polydipsia, polyuria, and weight loss, and 1 day of acute onset abdominal pain and emesis. She was thin (BMI 0.4^th^ percentile) with otherwise unremarkable physical examination. Initial labs showed hyperglycemia (1036 mg/dL), ketosis (beta-hydroxybutyrate 5.3 mmol/L), acidosis (pH 7.27), low insulin level (< 1.0), and low c-peptide (0.35 ng/mL). HTG was noted (1763 mg/dL), with normal lipase level (35 U/L). She tested positive for the diabetes autoantibodies GAD and ICA 512. Thyroid autoantibodies were negative. Family history was notable for hyperlipidemia in her maternal grandmother.

Fluid resuscitation was performed, followed by initiation of insulin infusion at 0.1 units/kg/h. Serial fasting TG levels improved to 189 mg/dL at 18 h after insulin initiation, at which point she transitioned to subcutaneous insulin. She did not require any additional antilipidemic agents. TG level was normal at 74 mg/dL 3 years later (age 9 years).

## 6. Case E

A previously healthy 5-year-old female presented to the ED in DKA due to new-onset T1D. Preceding symptoms included 2 weeks of weight loss (3.6 kg), polyuria, polydipsia, fatigue, acute vomiting, and abdominal pain. Physical examination was notable for tachypnea and dry mucous membranes with normal abdominal exam. BMI was at the 44^th^ percentile. Lab results confirmed DKA (glucose: 463 mg/dL, beta-hydroxybutyrate: 5.4 mmol/L, pH 7.15), and showed HTG (1562 mg/dL); A1C was 10.1%. She tested positive for the diabetes autoantibodies GAD 65, ICA 512, and insulin autoantibody. Thyroid autoantibodies were negative. Lipase level was not checked.

Fluid resuscitation was performed, followed by initiation of insulin infusion at 0.1 units/kg/h. She was transitioned to subcutaneous insulin at 14 h of hospitalization. At that time, repeat TG level was 217 mg/dL. Family history was notable for hyperlipidemia of unknown type in the patient's 11-year-old brother, mother, and maternal grandmother.

A lipid screening 5 years later (age 10 years) showed hypercholesterolemia (total cholesterol 291 mg/dL, low-density lipoprotein (LDL) 212 mg/dL) with normal TG level (56 mg/dL) and elevated A1c of 13.0%. Dietary and lifestyle modifications were advised with limited success. Repeat nonfasting lipid panel 2 years later (at 12 years of age) showed a total cholesterol of 298 mg/dL, LDL of 197 mg/dL, and triglycerides (TGs) of 145 mg/dL, prompting initiation of a high potency statin. Her diabetes remained uncontrolled at that time with A1c of 12.7%.

## 7. Discussion

We describe five cases of severe HTG in pediatric patients with new-onset T1D with DKA and provide short-term longitudinal data. Our single-center experience suggests that HTG may be an underrecognized entity in this population, as the majority of the presented cases were identified incidentally. Severe HTG can be associated with pancreatitis and this may require additional IV insulin therapy delaying transition to subcutaneous insulin. More studies are needed to guide appropriate lipid screening at time of pediatric T1D diagnosis. We suggest that screening for HTG in individuals with family history of hyperlipidemia, lipemic blood samples, protracted abdominal pain, and persistent acidemia may be warranted, as these factors may increase identification of severe HTG and guide timing of optimal transition from IV to subcutaneous insulin.

Although mild and transient HTG is seen in DKA (variable prevalence ranging from 30%–88%), severe HTG is extremely rare in adults (<1%) and has an unknown incidence in the pediatric T1D population. There are several case reports in the pediatric literature (Table 1). Richardson et al. [4] conducted the largest retrospective study on HTG by reviewing all causes of severe HTG in children over a 17-year-period (1999–2016); they reported 124 cases of severe HTG, of which ~19% were associated with T1D and 15% with DKA. The assessment of TG levels during the presentation of new-onset T1D (with or without DKA) is not the standard of care, so prevalence of severe HTG in new-onset T1D is unknown. Per the American Diabetes Association guidelines. a lipid panel is recommended soon after diagnosis, and after glycemic control is improved [24]. The cases presented here highlight the benefits of earlier lipid screening (before glycemic control improves), as earlier screening can recognize severe HTG, help effectively treat the condition and prevent HTG-induced complications.

Severe HTG (TG levels ≥ 1000 mg/dL) carries a 5% risk of acute pancreatitis in adults, and this risk rises to 10%–20% with TG ≥ 2000 mg/dL [3]. Severe HTG-induced acute pancreatitis can also be the first presentation of new-onset diabetes mellitus. The triad of pancreatitis, severe HTG, and DKA has been reported in the pediatric literature, but the incidence remains unknown [5–7, 9, 17]. Delayed initiation of pancreatitis treatment increases patient's morbidity and mortality risk as it can result in grave complications, such as shock, circulatory failure, severe respiratory distress, or multi-organ failure [7, 23]. Other severe HTG-related adverse effects include interference with the reliability of laboratory electrolyte measurements and HbA1c assays, and lipemia retinalis [11–13]. Concomitant presentation of DKA and pancreatitis presents a challenge for physicians, as a common symptom of DKA is abdominal pain, so pain due to pancreatitis may be falsely attributed to DKA. We suggest that for this reason, a lipase and TG should be checked in all patients who do not have full resolution of abdominal pain with resolution of acidosis. Further, due to the risk of lab interference we suggest HTG screening if acidosis measured on serum bicarbonate does not improve at a typical pace and the anion gap remains elevated despite ketosis resolution.

In our patients, insulin therapy combined with fasting was effective in reducing TG to < 1000 mg/dL. Most of the patients required continuous IV insulin infusion past DKA clearance, which has also been successful in previous published cases [14]. Three patients required over 30 h of intensive IV insulin therapy compared to the average of 15 h for uncomplicated severe DKA [20]. Limited data exist regarding typical insulin needs for pediatric HTG treatment, but in adults with diabetes an average of 55.8 h of IV insulin infusion was required to correct TG to less than 500 mg/dL [20]. All of our patients received continuous insulin infusion at a rate similar to what was published previously (0.1–0.3 U/kg/h) [1], with the exception of case C who initially required a lower rate of 0.05 due to hyperosmolar DKA. Notably, recent reports suggest that lower insulin infusion rates are equally effective in treatment of HTG in individuals without insulin resistance [24]. Although subcutaneous insulin can be used to treat severe HTG [1], we preferentially used IV insulin therapy to achieve rapid TG reduction. There was no clear association between initial level of HTG and presence of pancreatitis, age, or IV insulin requirement in our described cases (Table 2). This is consistent with previously published pediatric cases (Table 1). Plasma exchange is rarely used in the treatment of severe pediatric HTG, and was not required for any of the patients in this series [8, 19].

HTG in the setting of absolute insulin deficiency results from two mechanisms: (1) lipolysis activation in the adipose tissue resulting in increased free fatty acid (FFA) levels and accelerated formation of very-low-density lipoprotein (VLDL) by the liver, and (2) decreased LPL activity, resulting in reduced plasma clearance of VLDL and chylomicrons [1]. Although insulin deficiency is considered the primary reason for severe HTG in DKA, genetic predisposition for HTG may play a role, complicating the clinical picture. Previous research postulated that genotype variability affecting the *LPL* gene directs the degree of susceptibility to insulin deficiency-mediated severe HTG [11], however, most reports show transient HTG that does not require long-term lipid-lowering agents (Table 1). Only 4 out of the 15 patients reported in the literature received oral antilipidemic therapy during the acute phase of illness. While complete data are not available, at least 3 of the 15 cases in the literature remained on oral antilipidemic agents during outpatient follow-up. Similarly, two of the five patients in this report required antilipidemic treatment at the time of follow up, one early after initial treatment and one late in their care. Case A had resistant HTG requiring long-term fenofibrate treatment. Case E had recurrence of hyperlipidemia years later, raising concern that early and severe HTG may be associated with increased risk of HTG later in life. Both had strong family history of early onset hyperlipidemia suggesting a possible genetic component. Overall, it is clinically challenging to predict which individuals will require adjunct antilipidemic therapy, though we suspect that genetics play a role in some cases. Longitudinal data on the need for oral antilipidemic treatment in this population is sparse, and more research is needed to determine which individuals would benefit most from early or more frequent screening of TGs.

## 8. Conclusions

The cases presented highlight severe HTG as an early manifestation of new-onset pediatric T1D and its risk for complications. Early recognition of severe HTG allows for rapid TG clearance via prolongation of the fasting state and IV insulin therapy. Antilipidemic agents are required in select cases, but it is not clear which patients are at highest risk of requiring this treatment. The prevalence of severe HTG in cases of new-onset T1D remains unclear, as does the incidence of pancreatitis. Early detection of severe HTG in newly diagnosed T1D may reduce the risk for pancreatitis and aid in identifying patients who require outpatient antilipidemic therapy. We suggest a low threshold for screening for HTG in individuals with strong family history of hyperlipidemia, protracted abdominal pain, slow resolution of acidosis, or lipemic blood samples.

## Figures and Tables

**Table 1:** Summary of case-reports of patients with severe HTG and new-onset diabetes with their presentation characteristics.

Presentation characteristics	Patient 1 [5]	Patient 2 [13]	Patient 3 [6]	Patient 4 [11]	Patient 5 [7]	Patient 6 [10]	Patient 7 [8]	Patient 8 [9]	Patient 9 [14]	Patient 10 [15]	Patient 11 [12]	Patient 12 [16]	Patient 13 [21]	Patient 14 [22]	Patient 15 [23]
Age/sex	10 years/female	11 years/male	4 years/female	2.5 years/male	18 years/female	8 years/female	10 years/female	16 years/female	10 years/female	10 years/female	13 years/male	9 years/male	13 years/female	2 years/female	7 years/male

Initial clinical presentation	New-onset diabetes with DKA (pH 7.05)	New-onset T1D DKA (pH 7.26)	New-onset diabetes with DKA (pH 6.8)	New-onset diabetes with DKA (pH 7)	Acute pancreatitis	New-onset diabetes with DKA (pH 7)	New-onset diabetes with DKA (pH 6.87) and acute pancreatitis	New-onset diabetes with DKA (pH 6.8)	New-onset diabetes with DKA (pH 6.76) and acute pancreatitis	New-onset diabetes with DKA (pH 6.88)	New-onset diabetes with DKA (pH 6.98)	New-onset diabetes with DKA (pH 7.19)	New-onset diabetes with DKA (pH 7.10)	New-onset diabetes with DKA (7.1)	New-onset diabetes with DKA (pH60.87)

Reason to obtain TG	Acute pancreatitis	Lipemic blood sample	Lipemic blood sample	Unclear	Acute pancreatitis and lipemic blood sample	Lipemic blood sample	Lipemic blood sample	Lipemic blood sample	Lipemic blood sample	Lipemic blood sample	Lipemic blood sample	Lipemic blood sample	Lipemic blood sample	Lipemic blood sample	Lipemic blood sample

Comorbidities and complications	Acute pancreatitis (DOA 2)	—	Encephalopathy and acute pancreatitis (DOA1)	—	Circulatory shock (at 12 h) and discovery of DKA (pH 6.99) and new-onset diabetes	—	HTG resistant to 30 h of insulin therapy with decompensation to end-organ failure (acute kidney Injury and pleural effusion)	Acute pancreatitis (at 12 h)	Shock requiring pressor support (with 24 h)	—	—	—	Encephalopathy, eruptive xanthomas, and pancreatitis	—	Autism spectrum disorder and restrictive eating

Occurrence of acute pancreatitis	Yes	No	Yes	No	Yes	No	Yes	Yes	Yes	No	No	No	Yes	No	No

Acute severe HTG therapy during hospitalization	1. IV insulin (max 0.2 u/kg/h), hydration 2. Fasting	1. IV insulin (max 0.1 u/kg/h), hydration 2. Fasting	1. IV insulin, hydration 2.Fasting	1. IV insulin, hydration 2. Fasting 3. Partial plasmapheresis	1. IV insulin, hydration 2. Fasting	1. IV insulin, hydration 2. Fasting	1. IV insulin, hydration 2. Fasting 3. Plasmapheresis *⁣*^*∗*^ Fenofibrate initiated before discharge	1. IV insulin, hydration 2. Fasting *⁣*^*∗*^ Fenofibrate initiated before discharge	1. IV insulin (0.5–1.0 u/kg/h), hydration 2. Fasting 3. IV bicarbonate	1. IV insulin, hydration 2. Fasting	1. IV insulin (0.1 u/kg/h), hydration 2. Fasting	1. IV insulin (0.3 u/kg/h), hydration 2. Fasting 3. Heparin 4. Fenofibrate	1. IV insulin (0.1 u/kg/h), hydration 2. Fasting 3. Fenofibrate 4. Omega-4 fatty acid	1. IV insulin (0.1 u/kg/h), hydration 2. Reduced oral intake	1. IV insulin (0.124 u/kg/h), hydration 2. Fasting

Days to TG levels <1000	6 days	26 h	8 days	3 days	3 days	No data (5 days of hospitalization)	~ 2–3 days	48 h	96 h	Unclear (?3–8 days)	2 days	~ 3–5 days	8–9 days	2 days	7–8 days

Serum lipids on initial admission (md/dL)	TG: 8300 Total chol: 211	TG: 3573	TG: 13,846 Total chol: 1267	TG: 13,493 Total chol: 734	TG: 1724 Total chol: 752	TG: 10,850	TG: 16,334 Total chol: 467	TG: 930 2515 Total chol: 332	TG: 10,260 Total chol: 970	TG: 9991 Total chol: 1081	TG: 14,461 Total chol: ~1100	TG: 13,089 Total chol: 354	TG: 3540	TG: 11,470	TG: 17,675

Serum lipids upon discharge (md/dL)	TG: 420 Total chol: 346	TG: 644	TG: 675 Total chol: 555	TG: 766 Total chol: 337	TG: 283	No data	TG: <1000 (no value reported)	TG: 614	TG: 476	TG: 319 Total chol: 151	TG: 122 Total chol: 317	TG: 217	TG: 1187	No data	TG <1290

Serum lipids on latest outpatient follow-up (md/dL)	(at 2 months) TG: 96 Total chol: 188	(at 4 weeks) TG: 55 Total chol: 241	(at 1 month) TG: 90 Total chol: 110	(at 4 months) TG: 90 Total chol: 180	No data	(at 17 days) TG normalized (no value data)	TG normalized (no timing or value data)	(at 2 weeks) TG: 170	(at 2 months) TG: 82 Total chol: 173	After discharge (and up to 2year of follow up) and normal TG (no value data)	(at 6 month) TG: 96 Total chol: 164	(at 3 months) TG: 73	(at 1 month) TG: 65	(at 6 months) TG: 116	(at 2 weeks) TG: 75 (at 6 months) TG: 74

Outpatient lipid-lowering medication therapy and further testing	No medication	No medication	No medication	• Yes, transient statin therapy (total 2 weeks). • Genetic testing revealed heterozygous mutation of the LPL gene.	No data	No data	• Yes, transient use of fenofibrate. • Ruled out primary lipid metabolism disorder.	• Yes, fenofibrate • Undergoing evaluation for familial HC (due to presence of xanthomas)	No medication	• No medication. • Genetic testing revealed compound heterozygous for two mutations in the LPL gene.	No medication	• Unclear if continued on fenofibrate • Genetic testing planned	• Fenofibrate	• No medication • Genetic testing ruled out LPL mutation	• No medication

Abbreviations: Chol, cholesterol (mg/dL); DKA, diabetic ketoacidosis; DOA, date of admission; HC, hypercholesterolemia; HDL, high-density lipoprotein; HTG, hypertriglyceridemia; IV, intravenous; LDL, low-density lipoprotein; TG, triglycerides (mg/dL).

**Table 2:** Summary of cases reported in this series of children with HTG and new-onset diabetes.

Presentation characteristics	Case A	Case B	Case C	Case D	Case E
Age/sex	16 years/male	7 years/male	15 years/male	6 years/female	5 years/female
Initial clinical presentation	New-onset diabetes	New-onset diabetes with DKA	New-onset diabetes with DKA	New-onset diabetes with DKA	New-onset diabetes with DKA
Reason to obtain TG	Routine outpatient screening	Routine outpatient screening	Unknown	Unknown	Unknown
Comorbidities and complications	None	Cerebral edema	Altered mental status and shock	None	None
Occurrence of acute pancreatitis	No	No	Yes	No	No
Acute severe HTG therapy during hospitalization	1. Subcutaneous insulin 2. IV insulin (max 0.1 units/kg/h), hydration 3. Fasting	1. IV insulin (max 0.1 units/kg/h), hydration 2. Fasting	1. IV insulin (max 0.23 units/kg/h), hydration 2. Fasting	1. IV insulin (max 0.1 units/kg/h), hydration 2. Fasting	1. IV insulin (max 0.1 units/kg/h), hydration 2. Fasting
Days to TG levels <1000	7 days	2 days	1 day	1 day	1 day
Serum TG on initial admission (md/dL)	8486	6784	1981	1763	1562
Serum TG upon discharge (md/dL)	925	225	206	189	217
Serum lipids on latest outpatient follow-up (md/dL)	(at 16 months postdiagnosis) TG:120	(at 3 months postdiagnosis) TG:44	Not available	(at 3 years postdiagnosis) TG:74	(at 7 years postdiagnosis) TG:145
Outpatient lipid-lowering medication therapy and further testing	Fenofibrate and omega-3 acids	None	None	None	Statin
Family history of dyslipidemia	Yes	Yes	No	Yes	Yes

## Data Availability

The data that support the findings of this study are available upon request from the corresponding author.
